# Clinical Outcomes of Curettage *versus* Surgical Resection of Giant Cell Tumor of the Distal Radius – A Systematic Review and Meta-analysis

**DOI:** 10.1055/s-0044-1779321

**Published:** 2024-12-21

**Authors:** Sheikh Muhammad Ebad Ali, Syeda Safeena Fatima, Bisma Munawar, Maheen Fatima, Syeda Kisa Batool Naqvi, Laiba Urooj Malik

**Affiliations:** 1 Departamento de Emergência, Mamji Hospital, Karachi, Paquistão; 2 Karachi Medical and Dental College, Karachi, Paquistão; 3 Dow University of Health Sciences, Karachi, Paquistão; 4 Shaheed Mohtarma Benazir Bhutto Medical College, Karachi, Paquistão

**Keywords:** curettage, fracture, distal radius, giant cell tumor of bone, margins of excision, operative surgical procedures

## Abstract

**Objective** Surgical procedures of curettage and surgical resection are used to treat giant cell tumor (GCT) of the distal radius, but it is still controversial whether one provides better functional outcomes than the other. The present study aims to determine and compare both procedures regarding complications, local recurrence, and mobility.

**Methods** A complete search of the applicable literature was done and independently assessed by three authors. Included studies reported on patients who were surgically treated for GCT of the distal radius with either curettage or surgical resection. The Preferred Reporting Items for Systematic Reviews and Meta-Analysis (PRISMA) statement was used to obtain research regarding outcomes of surgical resection and curettage for GCT of the distal radius. A meta-analysis was performed using this data. Quality assessment was performed.

**Results** Seven studies, comprising 114 patients with resection and 108 with curettage, totaling 222 subjects with 117 males and 105 females, were included in the present review. Overall, patients in the curettage group had a higher recurrence rate (0.205; 95% confidence interval [95%CI] = 0.057–0.735; *p* = 0.015). Incidences in complications remains the same in both groups (2.845; 95%CI = 0.644–12.57; *p* = 0.168). Incidences in functional outcomes were the same in both groups as well (−0.948; 95%CI = −2.074–0.178; *p* = 0.099).

**Conclusion** The authors prefer resection and reconstruction for GCT of distal radius as optimum treatment method due to the similar functional outcomes and lower chances of recurrence. Curettage might be a treatment option in low-grade GCT coupled with adjuvant, neoadjuvant or ablation to reduce the risk of recurrence.

## Introduction

Giant cell tumor (GCT) of bone is a medullar tumor characterized by multinucleated cells conformable to osteoclasts usually of benign nature with the potential to be malignant. 1 The distal radius accounts for 10% of giant cell tumors of bone with a high rate of recurrence. 2 Its development leads to pain, swelling, tissue extension outwards to extremity; severe cases leading to joint deformity and disability if the joint is involved. Although GCT of the distal radius is not life threatening, it severely damages the bone and its surrounding tissues and makes it harder to have normal limb function. The treating surgeons ideally must work both on reducing the recurrence of the tumor and optimal mobility outcomes depending on the extent and nature of the tumor.

Surgical treatments for GCT of the distal radius involve curettage or surgical resection. 3 Adjuvants like liquid nitrogen, phenol, or cement are used to minimize tumor recurrence, although the use of adjuvants is still controversial. 4 The tumor has been classified by Campanacci et al. 5 based on the appearance of tumor on plain radiographs into three radiographic grades. From our literature review, we have not found systematic reviews primarily focusing on postoperative functional outcomes for GCT of the distal radius. Liu et al. 6 and Pazionis et al. 7 focused on the rate of recurrence and complications postoperatively while another recent review by Koucheki et al. 8 included functional outcomes, but only two of the included studies reported functional outcomes by functional restoration after resection and curettage for GCT of the distal radius. Therefore, a consistent approach is needed to systematically review the literature to compare the functional outcomes after resection and curettage for GCT of the distal radius.

The aim of the present study is to compare the functional outcomes of both surgical procedures, that is, curettage and surgical resection for GCT of the distal radius and come to a coherent conclusion as to which procedure gives us a better outcome in terms of function, complications, and recurrence.

## Materials and Methods

### Strategy

**Fig. 1:**
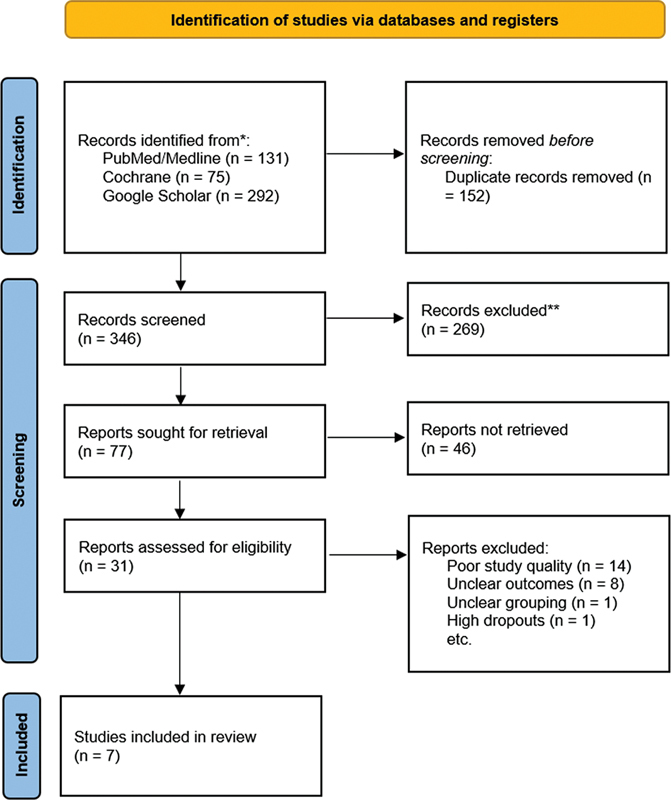
Preferred Reporting Items for Systematic Reviews and Meta-Analysis (PRISMA) chart showing the inclusion and exclusion of studies.

The Preferred Reporting Items for Systematic Reviews and Meta-Analyses (PRISMA) statement was used to obtain research regarding outcomes of surgical resection and curettage for GCT of the distal radius. The available literature was studied to ensure quality assessment scores. The inclusion and exclusion of studies are shown in Fig. 1 .

### Database

The PubMed/Medline, Google Scholar and Cochrane library were systematically searched from 1990 to 2019 with the MESH terms *resection* , *curettage* , *distal radius* and *giant cell tumor* in different combinations for comparative trials in English on human specimens. References of included trials were also checked for eligible studies.

### Inclusion and Exclusion Criteria

After discussions among the authors, the inclusion and exclusion criteria were determined. Only comparative studies including randomized trial and cohort studies that involved specified outcomes for surgical resection versus curettage for GCT of the distal radius were considered. The included participants in trials could not have any other associated bone tumors or malignant GCT. The interventions were limited to surgical resection or curettage, with or without adjuvant or neoadjuvant denosumab and zoledronate therapy to solidify the tumors. However, the trials including adjuvant radiotherapy and chemotherapy were not included. The other exclusion criteria to the study were: poor quality trials, letters, short communications, commentaries, editorials, case reports, single-armed studies, conference papers, proceedings, and personal communications. The corresponding author of the present article contacted the authors of trials to elucidate any other possible outcome in their study before exclusion, in the case of no response or undesired response.

### Risk of Bias and Quality Assessment

The research was independently scored by three authors (SMEA, LUM, and SSF)with the quality assessment checklist for methodological quality by the Oxford quality scoring system (OQSS) for randomized trials. 9 For the Oxford quality scoring system, a score of 5 or 4 suggests a good quality trial; 3 or 2 predicts an average-quality trial while, 1 or 0 signifies a poor-quality study. For nonrandomized comparative studies, the Modified Newcastle-Ottawa Scale was used, where above 7 stars are indicative of good quality trial, while 4–7 stars suggest a fair-quality trial and less than 4 stars signify a poor-quality trial. 10 Any disagreements were settled through internal discussion among the authors. An expert was involved if disagreements could not be resolved after discussions among authors. Table 1 shows the quality of included studies.

**Table 1:** Study characteristics of the studies included in the present review

Author	Year	Country	Study design	Total patients	Resection versus curettage	Reconstruction	Age	Gender – M: F	Follow-up	Quality	Campanacci grading	Neoadjuvant	Adjuvant
Cheng et al. 11	2001	Taiwan	Prospective comparative	12	6:6	Fibular graft	35 ± 14	4:8	72 ± 39	Fair	All grade III	None	Phenol, alcohol, saline*
Abuhejleh et al. 12	2020	Canada	Retrospective comparative	57	23:34	Bone cement / graft	35.4 ± 10	25:32	89 ± 69.75	Fair	Grade II = 13, Grade III= 40	None	Saline*
Sheth et al. 13	1995	USA	Comparative	22	11:11	Bone cement	34 ± 16	12:14	108 ± 93.5	Good	Grade I = 2, Grade II =8 Grade III =16	None	Cryosurgery*
Mozaffarian et al. 14	2018	Iran	Prospective comparative	13	7:6	Fibular arthroplasty	33.6 ± 5.25	6:7	72 ± 5	Fair	NA	None	Saline*
Wysocki et al. 15	2015	USA	Retrospective comparative	39	19:20	osteoarticular allograft	34 ± 10	22:17	132 ± 76.25	Fair	Grade II = 15, Grade III = 24	None	Phenol, Electrocautery, Argon beam, PMMA*
Jiao et al. 16	2021	China	Retrospective study	21	11:10	Fibular graft	36 ± 7.5	13:8	29 ± 9	Fair	Grade II = 11, Grade III = 10	None	Microwave ablation in curettage
Zou et al. 17	2019	China	Retrospective study	58	37:21	Fibular graft	33.2 ± 12.5	35:23	95.3 ± 75	Good	NA	Denosumab	PMMA in curettage

Abbreviations: F, Female; M, male; PMMA, polymethyl methacrylate; USA, United States of America.

Note: *In both groups

### Data Extraction

The data extracted from each of the study by authors (SSF, MF, LUM, KN, and BMZ) were year of publication, country of the study, study design, participants in total and in each group, gender, age, Campanacci grade, follow-ups, functional restoration, complications, and recurrences. The extracted data from the included studies is shown in Tables 1 and 2 . 11 12 13 14 15 16 17

**Table 2:** Outcomes of included studies

Author	Functional restoration in resection	Functional restoration in curettage	Complications in resection	Complications in curettage	Recurrence in resection	Recurrence in curettage
Cheng et al. 11	NA	NA	2	1	1	1
Abuhejleh et al. 12	NA	NA	7	0	1	10
Sheth et al. 13	54.33 ± 12.33	65 ± 22.33	4	8	0	3
Mozaffarian et al. 14	67.17 ± 6.08	78.67 ± 5.77	1	0	0	4
Wysocki et al. 15	67.46 ± 12.55	54.29 ± 16.07	3	1	1	12
Jiao et al. 16	45.66 ± 2.78	54.83 ± 3.73	0	0	0	1
Zou et al. 17	55.66 ± 12.67	67.33 ± 11.33	12	0	10	5

Abbreviation: NA, not available.

### Outcomes

Musculoskeletal tumor society scores (MSTS), Mayo wrist score (MWS), Visual analogue score (VAS), and Disabilities of the Arm, Shoulder, and Hand (DASH) were the commonly employed scores in different studies. The functional restoration was assessed as primary outcome by a unique methodology to overcome the high level of disparity seen among the scores used for reporting outcomes in individual studies. We measured the mean ± standard deviation (SD) of the reported percentage of wrist mobility including pronation, supination, flexion, extension, and grip strength compared with the unaffected wrist. The secondary outcomes were incidence of surgery-related complications and recurrences as shown in Table 2 .

### Statistical Analysis

The data analysis was designed by two authors (SMEA and SSF). The data were analyzed by authors (SMEA, MF and BMZ) using SPSS Statistics for Windows, version 23.0 (IBM Corp., Armonk, NY, USA). Mean ± SD values were used to express the continuous variables whereas the categorical variables were expressed as numbers, and the odds ratio (OR) was used to pool the estimate with a 95% confidence interval (95%CI) in the forest plots. A 2 × 2 table was drawn up where categorical data was plotted. The OpenMetaAnalyst Software was used to draw up the forest plots of the outcomes, using the random-effects, generic inverse variance method of DerSimonian and Laird. A random-effects model with a 95%CI was used to pool the OR of complications and recurrences after resection or curettage, while the standardized mean difference (SMD) was used to pool the estimates for the functional restoration. The heterogeneity was assessed by I ^2^ Statistics. The heterogeneity was considered negligible when there was an I ^2^ < 25%; low when there was an I ^2^ of 26–50%; moderate when there was an I ^2^ of 51–75%; and high, when there was an I ^2^ > 75%. The assessment of the statistically significant moderate or high between-study heterogeneity (I ^2^ > 50%; *p* < 0.05) for primary outcomes was made by conducting the random-effect meta-regression to forecast the factors affecting the success and failure of the intervention in GCT of the distal radius. The publication bias will be assessed by the funnel plot and Egger and Begg tests if ten or more studies fulfil the inclusion criteria.

## Results

### Study Characteristics

During the literature search from databases, we identified 131 studies from PubMed/MEDLINE, 75 studies from Cochrane, and 292 studies from Google Scholar. The studies were screened by titles and 346 duplicate studies were removed. During the abstract screening of 152 articles after duplicate removal, 121 articles were excluded, while full texts of 31 studies were reviewed for eligibility according to the inclusion and exclusion criteria. A total of 24 studies were excluded after reading the full text due to ineligibility, poor methodology, unclear outcomes, high rate of dropouts and ambiguous grouping.

Seven studies, comprising 114 patients with resection and 108 with curettage, totaling 222 subjects with 117 males and 105 females, were included in the present review, as shown in Table 1 . The studies were based in Taiwan ( *n* = 1), Iran ( *n* = 1), China ( *n* = 2), Canada ( *n* = 1), and United States ( *n* = 2). Two studies were of good quality and five studies were of fair quality. The means of age and follow-up in months of the candidates in the included studies were 22.08 ± 7.95 years and 71.57 ± 17.47 months, respectively.

### Functional Restoration

**Fig. 2:**
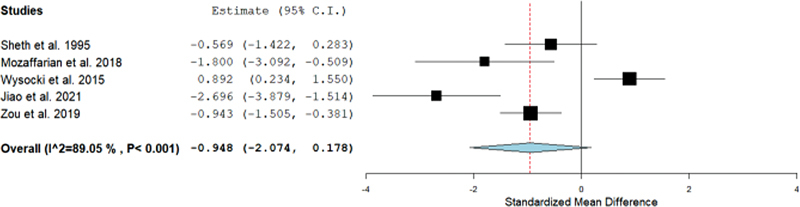
Forest plot showing the standardized mean difference (SMD) estimates for the functional restoration after resection versus curettage, in which the boxes show the effect size, with the length of the corresponding line explaining the 95% confidence interval (95%CI) and the diamond-shaped symbol representing the overall effect size.

The primary outcomes of our systematic review focused on the functional restoration of the wrist joint among resection and curettage groups. The difference in SMD between resection and curettage remained insignificant with −0.948 (95%CI= −2.074–0.178; *p* = 0.099) with statistically significant heterogeneity (I ^2^ = 89.05%; *p* < 0.001) as shown in Fig. 2 . Therefore, the functional outcomes were the same between both groups.

**Fig. 3:**
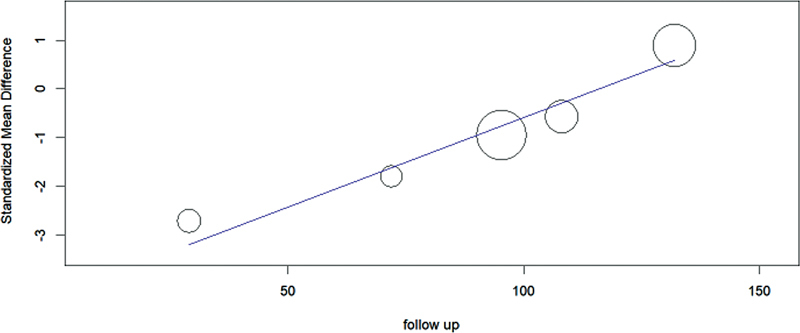
Scatter plot showing the standardized mean difference (SMD) estimates for the functional restoration after resection versus curettage on y-axis and follow-up duration on x-axis, in which the circles show the effect size of studies, with the slope of the line explaining the overall trend.

Meta regressions were performed to evaluate the cause of heterogeneity. A regression analysis was performed for each covariate to analyze the effect on I ^2^ individually. Duration of follow-up was found to be statistically significant ( *p* < 0.001) on meta regression, as shown in Fig. 3 .

### Complications

**Fig. 4:**
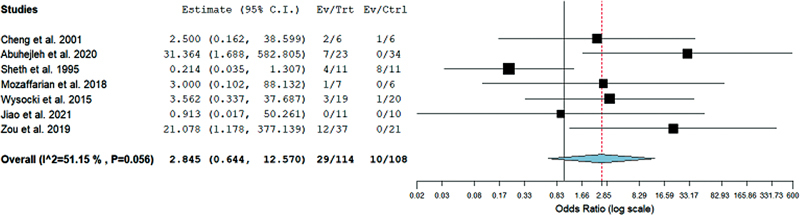
Forest plot showing the odds ratio (OR) estimates for the incidence of complications after resection versus curettage, in which the boxes show the effect size, with the length of the corresponding line explaining the 95%CI and the diamond-shaped symbol representing the overall effect size.

We assessed the rate of complications among the resection and curettage groups. The difference in OR between resection and curettage remained insignificant with 2.845 (95%CI = 0.644–12.57; *p* = 0.168) with statistically insignificant heterogeneity (I ^2^ = 51.5%; *p* = 0.056), as shown in Fig. 4 . Hence, the incidence of complications remains the same in both groups.

### Recurrence

**Fig. 5:**
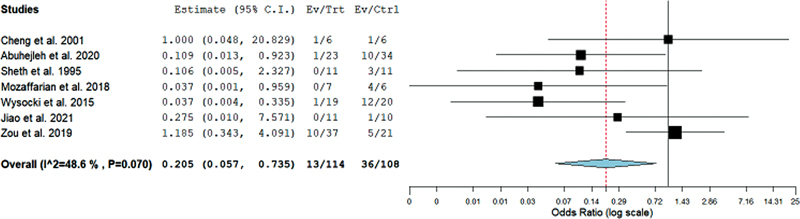
Forest plot showing the odds ratio (OR) estimates for the incidence of recurrences after resection versus curettage, in which the boxes show the effect size, with the length of the corresponding line explaining the 95%CI and the diamond-shaped symbol representing the overall effect size.

Another outcome of our systematic review was the incidence of recurrence among the resection and curettage groups. The difference in OR between resection and curettage remained significant with 0.205 (95%CI = 0.057–0.735; *p* = 0.015) with statistically insignificant heterogeneity (I ^2^ = 48.6%; *p* = 0.07) as shown in Fig. 5 . Therefore, the rate of recurrence was higher in the curettage group.

## Discussion

Giant cell tumor has been a benign tumor where surgical options with curative intents have been utilized since decades. However, denosumab and zoledronic acids have been recently employed as neoadjuvant and adjuvant chemotherapeutic agents in a number of trials where the postoperative results are controversial. 18 19 20 21 22 Surgical options are of two types; wide margin resection and curettage. 23 24 Wide margin resection requires extensive removal of bone with 2 to 5 cm of normal bone margins, while in curettage a small holed curettage is performed intralesionally without extensive bone loss. 24 During our literature review, we found that certain studies favored wide margin resection for Campanacci grade III GCT while curettage is preferred for Campanacci grade I and II GCT. 6 25 26 Most of the reviews considered different functional scores which lead to difference of opinions in their results. 7 Therefore, we also devised a method where we considered the average restoration of movements compared with the unaffected site. This led to inclusion of studies which reported functional outcomes by MSTS, MWS, DASH, or VAS.

The distal radius is the most common site for GCT. 11 However, it remains a difficult surgical site due to the biomechanical role of the distal radius in the mobility of the wrist joint. Distal radius forms radioulnar, radioscaphoid, and radiolunate joints while also forming the triangular fibrocartilage complex, radial collateral, radiocarpal and radioulnar ligaments with surrounding distal ulna and carpal. 27 These structures allow flexion, extension, radial deviation, ulnar deviation, supination, and pronation of the wrist joint with stability. 27 Therefore, care during distal radius surgery is needed to maintain the biomechanical stability of the wrist joint. However, from our systematic review, the functional restoration remains the same in each group whether resection or curettage was employed, contrary to Koucheki et al., 8 who regarded curettage as better for improved functional outcomes. A trend was observed in meta regression that with further follow-ups, the functional outcomes improved in the resection group. Therefore, we may consider that curettage may offer better outcomes earlier than resection but eventually, with the passage of time, the results would be equivocal in both groups. The comparability of functional outcomes between resection and curettage have been shown in certain studies carried on GCT of other regions. 28 29

From our review, the complications arising in each group were in a similar trend with insignificant heterogeneity. The complications considered were postoperative infections, arthritis, fractures, contractures, and graft rejection. The results of our review are contrary to the published studies and reviews where resections are considered as procedures with higher complications. 3 8 26 30 31 The results also showed that there are no increased risks of postoperative fractures in patients with curettage. 32 Complications during resection may be minimized by employing proper surgical techniques and experienced handling of the tissues, while during curettage, proper filling of the bone defect with bone graft or bone cement warrants lower risk of fractures. Surgical complications arising in the wrist joint strongly affects the social activities of patients. Hence, surgical management of GCT of the distal radius requires the least risk of complications.

Recurrence has been a controversial point in treatment of GCT. Our study evaluates that risk of recurrence is higher with curettage than resection. The results are explained by the higher chances of positive margins after curettage than resection. 33 Newer techniques where neoadjuvant denosumab was thought to downstage the GCT to make curettage feasible have also shown increased risk of recurrence. 34 35 36 However, denosumab has shown some utility postoperatively after curettage in reducing the risk of recurrences. 35 Intraoperative cryotherapy and ablation have also come up as a measure to reduce the risk of recurrence. 37 Previous literature has also shown that curettage presents an increased risk of recurrences. 6 7 8 The revision surgery after recurrence in distal radius makes the patient liable to unwanted psychosocial effects as well. 38

The literature has shown different reconstructive techniques after resection that we have mentioned in Table 1 . We broadly classify the reconstructive methods into two: arthroplasty and arthrodesis. Arthroplasty is a joint preserving technique where joint mobility is allowed, and all the studies included in the present review showed the utility of auto graft or allograft from the proximal fibula. The other technique, called arthrodesis, involves fusion of the wrist where radio metacarpal fusion is created such that the patient can pick heavy loads. The studies included in our review have either used radiocarpal fusion by bone cements with pins or radio metacarpal fusion by fibular strut graft with locking compression plates (LCPs) or dynamic compression plates (DCPs) for arthrodesis. However, the literature has also shown ulnar centralization, ulnar translocation, and vacant space fixation as useful techniques. 39 Another concept arises with vascularized and nonvascularized bone graft where studies have shown variable results. From a theoretical perspective, vascularized bone grafts carry nutrients and blood supply to the bone, so the regenerative capability increases, which decreases the healing time as well. 40

There were certain limitations in our review. Firstly, we did not find randomized controlled trials. Secondly, we did not search the grey literature. Thirdly, publication bias was not assessed due to inclusion of < 10 studies. We also did not focus upon the functional outcomes after different reconstruction techniques after resection and curettage which includes arthrodesis, arthroplasty, bone cementing, and bone grafting. Jalan et al. reported the different reconstruction methods recently in their review. 30

## Conclusion

In conclusion, the authors prefer resection and reconstruction for GCT of the distal radius as the optimum treatment method due to the similar functional outcomes and lesser chance of recurrence, especially for high grade GCT. However, curettage might be a treatment option in low grade GCT coupled with additional steps such as adjuvant, neoadjuvant, or ablation to reduce the risk of recurrence. Earlier restoration of normal functions may also be achieved with curettage. Hence, proper selection of patients and surgical expertise must be kept in equation before making surgical decisions. The review also emphasizes the need of randomized prospective large sample sized studies regarding GCT of the distal radius to elucidate the outcomes after resection and curettage for GCT of the distal radius.
