# Supplementary material for: Clinical Outcomes of Curettage versus Surgical Resection of Giant Cell Tumor of the Distal Radius – A Systematic Review and Meta-analysis
Source: Rev Bras Ortop (Sao Paulo). 2024 Dec 21;59(6):e830–8. doi: 10.1055/s-0044-1779321 (PMC11663078; doi:10.1055/s-0044-1779321)
Supplement: Related texts

# Research article: Resultados clínicos da curetagem *versus* ressecção cirúrgica do tumor de células gigantes do rádio distal: Uma revisão sistemática e metanálise

**Keywords:** curetagem, fraturas distais do rádio, margens de excisão, procedimentos cirúrgicos operatórios, tumor de células gigantes do osso

## Abstract

**Objetivo** A curetagem e a ressecção cirúrgica são utilizadas para tratamento do tumor de células gigantes (TCG) do rádio distal, mas ainda há controvérsias acerca da superioridade de uma destas técnicas em relação à outra. O objetivo do presente estudo foi determinar e comparar os dois procedimentos quanto a complicações, recidiva local e mobilidade.

**Métodos** Três autores fizeram uma busca completa da literatura pertinente e a avaliaram de forma independente. Os estudos incluídos relataram pacientes submetidos ao tratamento cirúrgico do TCG do rádio distal por curetagem ou ressecção cirúrgica. A declaração dos Principais Itens para Relatar Revisões Sistemáticas e Metanálises (Preferred Reporting Items for Systematic Reviews and Meta-Analyses, PRISMA, em inglês) foi usada para determinar os desfechos da ressecção cirúrgica e curetagem do TCG do rádio distal. Estes dados foram a base de uma metanálise. Uma avaliação de qualidade também foi realizada.

**Resultados** A presente revisão incluiu 7 estudos, compreendendo 114 pacientes submetidos à ressecção e 108 à curetagem, totalizando 222 indivíduos (117 homens e 105 mulheres). De modo geral, os pacientes do grupo submetido à curetagem apresentaram maior taxa de recidiva (0,205; intervalo de confiança de 95% [IC95%] = 0,057–0,735; *p* = 0,015). A incidência de complicações foi a mesma em ambos os grupos (2,845; IC95% = 0,644–12,57; *p* = 0,168). As incidências de desfechos funcionais também foram as mesmas em ambos os grupos (−0,948; IC95% = −2,074–0,178; *p* = 0,099]).

**Conclusão** Os autores preferem a ressecção e reconstrução para tratamento do TCG do rádio distal como método ideal devido aos desfechos de resultados funcionais semelhantes e às menores chances de recidiva. A curetagem pode ser uma opção de tratamento no TCG de baixo grau, associada à terapia adjuvante, neoadjuvante ou ablação para redução do risco de recidiva.

## Introdução

O tumor de células gigantes (TCG) ósseo é um tumor medular caracterizado por células multinucleadas semelhantes a osteoclastos, geralmente de natureza benigna, mas com possibilidade de transformação maligna. 1 O rádio distal é acometido em 10% dos casos ósseos de TCG, com alta taxa de recidiva. 2 Seu desenvolvimento causa dor, aumento de volume e exteriorização do tecido até as extremidades; casos graves provocam deformidade e incapacidade articular. Embora o TCG do rádio distal não seja uma ameaça à vida, danifica gravemente o osso e os tecidos circundantes e dificulta o funcionamento normal dos membros. Idealmente, os cirurgiões responsáveis pelo tratamento devem reduzir a recidiva do tumor e otimizar a mobilidade, dependendo da extensão e da natureza da neoplasia.

Os tratamentos cirúrgicos para TCG do rádio distal são curetagem ou ressecção cirúrgica. 3 Adjuvantes, como nitrogênio líquido, fenol ou cimento ortopédico, são usados para minimizar a recidiva tumoral, embora tal prática ainda seja controversa. 4 O tumor foi classificado por Campanacci, 5 com base em sua aparência em radiografias simples em três graus. Em nossa revisão da literatura, não encontramos revisões sistemáticas com enfoque principalmente nos desfechos funcionais pós-operatórios do TCG do rádio distal. Liu et al. 6 e Pazionis et al. 7 analisaram a taxa de recidiva e complicações no período pós-operatório. Outra revisão recente, de Koucheki et al., 8 incluiu desfechos funcionais, mas apenas dois dos estudos incluídos relataram tais desfechos de acordo com o restauro funcional após a ressecção e curetagem do TCG do rádio distal. Portanto, é necessária uma abordagem consistente para revisar sistematicamente a literatura e comparar os desfechos funcionais após a ressecção e curetagem do TCG do rádio distal.

O objetivo do presente estudo foi comparar os desfechos funcionais de ambos os procedimentos cirúrgicos, ou seja, curetagem e ressecção cirúrgica para tratamento do TCG do rádio distal e chegar a uma conclusão coerente sobre qual procedimento leva ao melhor desfecho em termos de função, complicações e recorrência.

## Materiais e Métodos

## Estratégia

A declaração dos Principais Itens para Relatar Revisões Sistemáticas e Metanálises (Preferred Reporting Items for Systematic Reviews and Meta-Analyses, PRISMA, em inglês) foi utilizada para pesquisar os desfechos da ressecção cirúrgica e curetagem para tratamento do TCG do rádio distal. A literatura disponível foi estudada para assegurar a avaliação de qualidade. A inclusão e exclusão de estudos são mostradas na Fig. 1 .

**Fig. 1:** Diagrama da declaração dos Principais Itens para Relatar Revisões Sistemáticas e Metanálises (Preferred Reporting Items for Systematic Reviews and Meta-Analyses, PRISMA, em inglês) que mostra a inclusão e exclusão de estudos.

## Banco de Dados

Os bancos de PubMed/Medline, Google Scholar e Cochrane Library foram sistematicamente pesquisados de 1990 a 2019 com os termos MESH *ressecção* , *curetagem* , *radio distal* e *tumor de células gigantes* em diferentes combinações para obtenção de estudos comparativos sobre seres humanos em língua inglesa. As referências dos artigos incluídos também foram analisadas para determinação de estudos elegíveis.

## Critérios de Inclusão e Exclusão

Os critérios de inclusão e exclusão foram determinados por discussões entre os autores. Foram considerados apenas estudos comparativos, incluindo ensaios randomizados e estudos de coorte, com desfechos específicos para ressecção cirúrgica ou curetagem para tratamento do TCG do rádio distal. Os participantes incluídos nos estudos não poderiam ter quaisquer outros tumores ósseos associados ou TCG maligno. As intervenções limitaram-se à ressecção cirúrgica ou curetagem, com ou sem terapia adjuvante ou neoadjuvante com denosumabe e zoledronato para solidificação dos tumores. No entanto, os estudos incluindo radioterapia e quimioterapia adjuvantes não foram incluídos. Outros critérios de exclusões foram estudos de baixa qualidade, cartas, comunicações curtas, comentários, editoriais, relatos de casos, estudos de braço único, documentos de conferências, anais e comunicações pessoais. O autor correspondente do presente artigo contatou os autores dos artigos para elucidação de qualquer outro desfecho possível antes da exclusão, no caso de ausência de resposta ou resposta indesejada.

## Risco de Viés e Avaliação de Qualidade

A pesquisa foi pontuada de forma independente por três autores (SMEA, LUM e SSF) de acordo com a lista de verificação de avaliação de qualidade metodológica Oxford quality scoring system (OQSS, em inglês) para estudos randomizados. 9 Neste sistema, a pontuação de 5 ou 4 sugere um estudo de boa qualidade; 3 ou 2 prevê um estudo de qualidade moderada, enquanto 1 ou 0 indica um estudo de baixa qualidade. Os estudos comparativos não randomizados foram avaliados de acordo com a Modified Newcastle-Ottawa Scale. Nesta escala, a pontuação acima de sete estrelas indica um estudo de boa qualidade, enquanto quatro a sete estrelas sugerem um ensaio de qualidade moderada e menos de quatro estrelas significa um ensaio de baixa qualidade. 10 Eventuais divergências foram resolvidas por meio de discussão interna entre os autores. Um especialista era chamado caso as divergências não pudessem ser resolvidas após as discussões entre os autores. A Tabela 1 mostra a qualidade dos estudos incluídos.

**Tabela 1:** Características dos estudos incluídos na revisão

Autor	Ano	País	Delineamento experimental	Total de pacientes	Ressecção *versus* curetagem	Reconstrução	Idade	Gênero – M:F	Acompanhamento	Qualidade	Grau de Campanacci	Neoadjuvante	Adjuvante
Cheng et al. 11	2001	Taiwan	Prospectivo comparativo	12	6:6	Enxerto fibular	35 ± 14	4:8	72 ± 39	Moderada	Todos grau III	Não	Fenol, álcool, salina*
Abuhejleh et al. 12	2020	Canadá	Retrospectivo comparativo	57	23:34	Cimento ortopédico/enxerto	35,4 ± 10	25:32	89 ± 69,75	Moderada	Grau II = 13, Grau III= 40	Não	Salina*
Sheth et al. 13	1995	EUA	Comparativo	22	11:11	Cimento ortopédico	34 ± 16	12:14	108 ± 93,5	Boa	Grau I = 2, Grau II =8 Grau III =16	Não	Criocirurgia*
Mozaffarian et al. 14	2018	Irã	Prospectivo comparativo	13	7:6	Artroplastia fibular	33,6 ± 5,25	6:7	72 ± 5	Moderada	NA	Não	Salina*
Wysocki et al. 15	2015	EUA	Retrospectivo comparativo	39	19:20	Aloenxerto osteoarticular	34 ± 10	22:17	132 ± 76,25	Moderada	Grau II = 15, Grau III = 24	Não	Fenol, eletrocauterização, feixe de argônio, PMMA*
Jiao et al. 16	2021	China	Retrospectivo	21	11:10	Enxerto fibular	36 ± 7,5	13:8	29 ± 9	Moderada	Grade II = 11, Grade III = 10	Não	Ablação por micro-ondas na curetagem
Zou et al. 17	2019	China	Retrospectivo	58	37:21	Enxerto fibular	33,2 ± 12,5	35:23	95,3 ± 75	Boa	NA	Denosumabe	PMMA na curetagem

Abreviaturas: EUA, Estados Unidos da América; F, Feminino; M, masculino; PMMA, polimetilmetacrilato.

Nota: *Nos dois grupos.

## Extração de Dados

Os dados extraídos de cada estudo pelos autores (SSF, MF, LUM, KN e BMZ) foram ano de publicação, país do estudo, delineamento experimental, número de participantes no total e em cada grupo, gênero, idade, grau de Campanacci, acompanhamentos, restauro funcional, complicações e recidivas. Os dados extraídos dos estudos incluídos são mostrados nas Tabelas 1 e 2 . 11 12 13 14 15 16 17

**Tabela 2:** Desfechos dos estudos incluídos

Autor	Restauro funcional após a ressecção	Restauro funcional após a curetagem	Complicações após a ressecção	Complicações após a curetagem	Recidiva após a ressecção	Recidiva após a curetagem
Cheng et al. 11	ND	ND	2	1	1	1
Abuhejleh et al. 12	ND	ND	7	0	1	10
Sheth et al. 13	54,33 ± 12,33	65 ± 22,33	4	8	0	3
Mozaffarian et al. 14	67,17 ± 6,08	78,67 ± 5,77	1	0	0	4
Wysocki et al. 15	67,46 ± 12,55	54,29 ± 16,07	3	1	1	12
Jiao et al. 16	45,66 ± 2,78	54,83 ± 3,73	0	0	0	1
Zou et al. 17	55,66 ± 12,67	67,33 ± 11,33	12	0	10	5

Abreviatura: ND, não disponível.

## Desfechos

As pontuações do Musculoskeletal Tumor Society Score (MSTS, em inglês), Mayo Wrist Score (MWS, em inglês), Escala Visual Analógica (EVA) e Disabilities of the Arm, Shoulder, and Hand (DASH, em inglês) foram comumente empregados em diferentes estudos. O restauro funcional foi avaliado como resultado primário por uma metodologia única para superar o alto nível de disparidade observado entre as pontuações utilizadas para relato de desfechos em cada estudo. Determinamos a média ± desvio padrão (DP) da porcentagem relatada de mobilidade do punho, incluindo pronação, supinação, flexão, extensão e força de preensão em comparação ao punho não acometido. Os desfechos secundários foram a incidência de complicações relacionadas à cirurgia e recidivas e são mostrados na Tabela 2 .

## Análise Estatística

A análise dos dados foi projetada por dois autores (SMEA e SSF). Os dados foram analisados pelos autores (SMEA, MF e BMZ) no programa IBM SPSS Statistics for Windows, versão 23.0 (IBM Corp., Armonk, NY, Estados Unidos). Variáveis contínuas foram expressas como valores de média ± DP. Variáveis categóricas foram expressas como números e a razão de chances ( *odds ratio* , OR, em inglês) agrupou a estimativa com intervalo de confiança de 95% (IC95%) em gráficos em floresta. Uma tabela 2 × 2 foi elaborada para plotagem dos dados categóricos. O programa OpenMetaAnalyst foi utilizado para traçar os gráficos em floresta dos desfechos segundo o método genérico de variância inversa de efeitos aleatórios de DerSimonian e Laird. Um modelo de efeitos aleatórios com IC95% agrupou a OR de complicações e recidivas após a ressecção ou curetagem, enquanto a diferença média padronizada (DMP) agrupou as estimativas de restauro funcional. A heterogeneidade foi testada por I ^2^ . A heterogeneidade foi considerada insignificante quando I ^2^ foi inferior a 25%; baixa quando I ^2^ variou entre 26 e 50%; moderada quando I ^2^ foi de 51 a 75%; e alta quando I ^2^ ficou acima de 75%. A avaliação da heterogeneidade moderada ou alta estatisticamente significativa entre os estudos (I ^2^ > 50%, *p* < 0,05) de desfechos primários foi feita por metarregressão de efeito aleatório para prever os fatores que afetam o sucesso e o fracasso da intervenção para tratamento do TCG do rádio distal. O viés de publicação seria avaliado por gráfico de funil e testes de Egger e Begg se dez ou mais estudos preenchessem os critérios de inclusão.

## Resultados

## Características do Estudo

À busca de literatura nos bancos de dados, identificamos 131 estudos no PubMed/MEDLINE, 75 estudos na Cochrane Library e 292 estudos no Google Scholar. Os estudos foram escolhidos por títulos e 346 estudos duplicados foram removidos. Durante a triagem dos resumos de 152 artigos após a remoção de duplicatas, 121 artigos foram excluídos, enquanto os textos completos de 31 estudos foram revisados quanto à elegibilidade de acordo com os critérios de inclusão e exclusão. Vinte e quatro estudos foram excluídos após a leitura do texto completo devido à inelegibilidade, má metodologia, resultados pouco claros, alta taxa de desistência e agrupamento ambíguo.

Sete estudos, compreendendo 114 pacientes submetidos à ressecção e 108 à curetagem, totalizando 222 indivíduos (117 do sexo masculino e 105 do sexo feminino), foram incluídos na presente revisão, como mostra a Tabela 1 . Os estudos foram realizados em Taiwan ( *n* = 1), Irã ( *n* = 1), China ( *n* = 2), Canadá ( *n* = 1) e Estados Unidos ( *n* = 2). Dois estudos eram de boa qualidade e cinco estudos tinham qualidade razoável. As médias de idade e o período de acompanhamento dos participantes dos estudos incluídos foram 22,08 ± 7,95 anos e 71,57 ± 17,47 meses, respectivamente.

## Restauro Funcional

Os desfechos primários da nossa revisão sistemática enfocaram o restauro funcional da articulação do punho entre os grupos submetidos à ressecção ou curetagem. A DMP entre ressecção e curetagem permaneceu insignificante, em −0,948 (IC95% = −2,074–0,178; *p* = 0,099) com heterogeneidade estatisticamente significativa (I ^2^ = 89,05%; *p* < 0,001), como mostra a Fig. 2 . Portanto, os desfechos funcionais foram iguais entre os dois grupos.

**Fig. 2:** Gráfico em floresta que mostra as estimativas de diferença média padronizada do restauro funcional após a ressecção ou curetagem. As caixas mostram o tamanho do efeito e o comprimento da linha correspondente indica o intervalo de confiança de 95% (IC95%). O símbolo em forma de diamante representa o tamanho geral do efeito.

Metarregressões foram realizadas para avaliar a causa da heterogeneidade. Uma análise de regressão foi feita para cada covariável para analisar o efeito individual sobre I ^2^ . A metarregressão mostrou que as durações do acompanhamento foram estatisticamente significativas ( *p* < 0,001), como ilustra a Fig. 3 .

**Fig. 3:** Gráfico de dispersão que mostra as estimativas da diferença média padronizada do restauro funcional após a ressecção ou curetagem no eixo y e a duração do acompanhamento no eixo x. Os círculos indicam o tamanho do efeito dos estudos e a inclinação da linha mostra a tendência geral.

## Complicações

Avaliamos a taxa de complicações entre o grupo submetido à ressecção ou curetagem. A diferença na razão de chances entre ressecção e curetagem continuou insignificante, em 2,845 (IC95% = 0,644–12,57; *p* = 0,168], assim como a heterogeneidade (I ^2^ = 51,5%; *p* = 0,056), como mostra a Fig. 4. Assim, a incidência de complicações foi a mesma em ambos os grupos.

**Fig. 4:** Gráfico em floresta que mostra as estimativas de razão de chances (OR) da incidência de complicações após a ressecção ou curetagem. As caixas mostram o tamanho do efeito e o comprimento da linha correspondente indica o IC95%. O símbolo em forma de diamante representa o tamanho geral do efeito.

## Recidiva

Outro desfecho da nossa revisão sistemática foi a incidência de recidiva entre os grupos submetidos à ressecção e curetagem. A diferença de OR entre ressecção e curetagem foi significativa, de 0,205 (IC95% = 0,057–0,735 *;* *p* = 0,015) com heterogeneidade estatisticamente insignificante (I ^2^ = 48,6%; *p* = 0,07), conforme mostrado na Fig. 5 . Portanto, a taxa de recidiva foi maior no grupo submetido à curetagem.

**Fig. 5:** Gráfico em floresta que mostra as estimativas de razões de probabilidade (OR) da incidência de recidivas após a ressecção ou curetagem. As caixas mostram o tamanho do efeito e o comprimento da linha correspondente indica o IC95%. O símbolo em forma de diamante representa o tamanho geral do efeito.

## Discussão

O TCG é um tumor benigno, cujas opções cirúrgicas com fins curativos são utilizadas há décadas. Entretanto, o denosumabe e o ácido zoledrônico foram recentemente empregados como agentes quimioterápicos neoadjuvantes e adjuvantes em vários estudos com desfechos pós-operatórios controversos. 18 19 20 21 22 Há duas opções cirúrgicas: a ressecção de margem ampla e a curetagem. 23 24 A ressecção de margem ampla requer a remoção extensa de osso, com 2 a 5 cm de margens ósseas normais; a curetagem com pequeno orifício é realizada de forma intralesional sem perda óssea extensa. 24 Durante nossa revisão de literatura, certos estudos favoreceram a ressecção com margens amplas para tratamento do TCG de grau III de Campanacci, enquanto a curetagem é preferida no TCG de grau I e II de Campanacci. 6 25 26 A maioria das revisões considerou diferentes escores funcionais, o que levou a diferenças de opiniões nos desfechos. 7 Portanto, também desenvolvemos um método em que consideramos o restauro médio dos movimentos em comparação ao sítio não acometido. Isto levou à inclusão de estudos que relataram desfechos funcionais por MSTS, MWS, DASH ou EVA.

O rádio distal é o sítio mais acometido pelo TCG. 11 No entanto, este ainda é um sítio cirúrgico difícil devido ao papel biomecânico do rádio distal na mobilidade da articulação do punho. O rádio distal forma as articulações radioulnar, radioescafoide e radiolunar; além disso, também forma o complexo de fibrocartilagem triangular, ligamentos colaterais radiais, radiocárpicos e radioulnares com a ulna distal e o carpo circundantes. 27 Essas estruturas permitem flexão, extensão, desvio radial, desvio ulnar, supinação e pronação da articulação do punho com estabilidade. 27 Portanto, é necessário cuidado durante a cirurgia do rádio distal para manter a estabilidade biomecânica da articulação do punho. Porém, em nossa revisão sistemática, o restauro funcional foi o mesmo em cada grupo, independentemente da realização de ressecção ou curetagem. Isso difere de Koucheki et al. 8 , que consideraram que a curetagem levava a melhores desfechos funcionais. A metarregressão gerou uma tendência em que, com mais acompanhamentos, os desfechos funcionais melhorariam no grupo submetido à ressecção. Assim, podemos considerar que a curetagem talvez ofereça melhores desfechos antes da ressecção, mas, com o passar do tempo, os desfechos seriam ambíguos nos dois grupos. A comparabilidade dos desfechos funcionais entre a ressecção e a curetagem foi demonstrada em alguns estudos realizados em TCG de outras localizações. 28 29

Em nossa revisão, as complicações observadas em cada grupo seguiram uma tendência semelhante, com heterogeneidade insignificante. As complicações consideradas foram infecções pós-operatórias, artrite, fraturas, contraturas e rejeição de enxertos. Os resultados da nossa revisão contrariam os estudos e revisões publicados que consideram a ressecção um procedimento com maiores complicações. 3 8 26 30 31 Os resultados também mostraram que não há maiores riscos de fraturas pós-operatórias em pacientes submetidos à curetagem. 32 As complicações durante a ressecção podem ser minimizadas pelo emprego de técnicas cirúrgicas adequadas e pelo manuseio experiente dos tecidos; à curetagem, o preenchimento adequado do defeito ósseo com enxerto ósseo ou cimento ortopédico assegura o menor risco de fraturas. As complicações cirúrgicas na articulação do punho afetam muito as atividades sociais dos pacientes. Logo, o manejo cirúrgico do TCG do rádio distal requer menor risco de complicações.

A recidiva é um ponto controverso no tratamento do TCG. Segundo nosso estudo, o risco de recidiva é maior com a curetagem do que com a ressecção. Os resultados são explicados pelas maiores chances de margens positivas após a curetagem do que a ressecção. 33 Técnicas mais recentes, acreditando que o denosumabe neoadjuvante reduziria o estágio do TCG para tornar a curetagem viável, também demonstraram maior risco de recidiva. 34 35 36 No entanto, o denosumabe teve certa utilidade pós-operatória nos casos de curetagem na redução do risco de recidivas. 35 A crioterapia intraoperatória e a ablação também surgiram como medidas para reduzir o risco de recidivas. 37 A literatura anterior também mostrou que a curetagem é associada a um maior risco de recidivas. 6 7 8 A cirurgia de revisão após a recidiva no rádio distal também sujeita o paciente a efeitos psicossociais indesejados. 38

A literatura mostrou diferentes técnicas reconstrutivas após a ressecção e as mencionamos na Tabela 1 . Os métodos reconstrutivos são classificados em duas categorias amplas: artroplastia e artrodese. A artroplastia é uma técnica de preservação articular em que a mobilidade articular é permitida e todos os estudos incluídos na presente revisão mostraram a utilidade do autoenxerto ou aloenxerto da fíbula proximal. A outra técnica, a artrodese, envolve a fusão do punho, criando uma fusão do rádio com o metacarpo para que o paciente possa suportar cargas pesadas. Os estudos incluídos em nossa revisão utilizaram fusão radiocárpica com cimentos ortopédicos e pinos ou fusão radiometacárpica por enxerto de haste fibular com placas de compressão de travamento ( *locking compression plate* , LCP, em inglês) ou placas de compressão dinâmica ( *dynamic compression plate* , DP, em inglês)para artrodese. Contudo, a literatura também relata a centralização ulnar, a translocação ulnar e a fixação de espaço vago como técnicas úteis. 39 Outro conceito é o enxerto ósseo vascularizado e não vascularizado, com desfechos variáveis. Do ponto de vista teórico, os enxertos ósseos vascularizados transportam nutrientes e suprimento sanguíneo para o osso, aumentando a capacidade regenerativa, o que também diminui o tempo de cicatrização. 40

Nossa revisão teve certas limitações. Em primeiro lugar, não encontramos estudos clínicos randomizados. Em segundo lugar, não pesquisamos a literatura cinzenta. Em terceiro lugar, o viés de publicação não foi avaliado devido à inclusão de menos de 10 estudos. Também não nos concentramos nos desfechos funcionais após diferentes técnicas de reconstrução depois da ressecção ou curetagem, que incluem artrodese, artroplastia, cimento ortopédico e enxerto ósseo. Jalan et al., recentemente, relataram os diferentes métodos de reconstrução em sua revisão. 30

## Conclusão

Concluímos que os autores preferem a ressecção do TCG e a reconstrução do rádio distal como método ideal de tratamento devido aos desfechos funcionais semelhantes e à menor chance de recidiva, em especial do TCG de alto grau. No entanto, a curetagem pode ser uma opção para tratamento do TCG de baixo grau, juntamente com outras etapas, como terapia adjuvante, neoadjuvante ou de ablação, para redução do risco de recidiva. O restauro precoce das funções normais também pode ser alcançado com a curetagem. Portanto, a seleção adequada de pacientes e a experiência cirúrgica devem ser consideradas antes de tomar decisões cirúrgicas. A revisão também enfatiza a necessidade de estudos prospectivos randomizados com grandes amostras de pacientes com TCG do rádio distal para elucidar os desfechos após sua ressecção ou curetagem.
